# Diffusion weighted MRI as an early predictor of tumor response to hypofractionated stereotactic boost for prostate cancer

**DOI:** 10.1038/s41598-018-28817-9

**Published:** 2018-07-10

**Authors:** David Pasquier, Abderraouf Hadj Henni, Alexandre Escande, Emmanuelle Tresch, Nick Reynaert, Olivier Colot, Eric Lartigau, Nacim Betrouni

**Affiliations:** 1 Academic Department of Radiation Oncology, Oscar Lambret Comprehensive Cancer Center, Lille, France; 2 CRIStAL laboratory, UMR CNRS 9189, University Lille 1, Villeneuve d’Ascq, France; 3 INSERM, U1171, University of Lille, Lille, France; 4 Department of Biostatistics, Oscar Lambret Comprehensive Cancer Center, Lille, France; 5 Department of Medical Physics, Oscar Lambret Comprehensive Cancer Center, Lille, France

## Abstract

We evaluated the feasibility of using the kinetic of diffusion-weighted MRI (DWI) and the normalized apparent coefficient diffusion (ADC) map value as an early biomarker in patients treated by external beam radiotherapy (EBRT). Twelve patients were included within the frame of a multicenter phase II trial and treated for intermediate risk prostate cancer (PCa). Multiparametric MRI was performed before treatment (M0) and every 6 months until M24. Association between nADC and PSA or PSA kinetic was evaluated using the test of nullity of the Spearman correlation coefficient. The median rates of PSA at the time of diagnosis, two years and four years after EBRT were 9.29 ng/ml (range from 5.26 to 17.67), 0.68 ng/ml (0.07–2.7), 0.47 ng/ml (0.09–1.39), respectively. Median nADC increased from 1.14 × 10^−3^ mm^2^/s to 1.59 × 10^−3^ mm^2^/s between M0 and M24. Only one patient presented a decrease of nADC (1.35 × 10^−3^ mm^2^/s and 1.11 × 10^−3^ mm^2^/s at M0 and M12 respectively). The increase in nADC at M6 was correlated with PSA decrease at M18, M24 and M30 (p < 0.05). The increase in nADc at M12 was correlated with PSA decrease at M36 (p = 0.019). Early nADC variation were correlated with late PSA decrease for patients with PCa treated by EBRT.

## Introduction

Prostate cancer (PCa) is the second most common cancer in men worldwide^1^. Radical prostatectomy, brachytherapy and external beam radiotherapy (EBRT) can be regarded as standard of care depending on risk stratification and patient characteristics^2,3^. Thus, a new challenge in PCa treatment is to predict the potential for relapse and toxicity in individual patients^4^. Many biomarkers or tests that predict treatment effectiveness, such as Oncotype DX, are in development^5^. Before treatment, some authors have studied classifications or nomograms for PCa, such as the d’Amico classification, to aid decision-making^6^.

After an EBRT procedure, guidelines recommend a follow-up based on prostate specific antigen (PSA) and clinical examination^7^. PSA nadir is often obtained 12 to 18 months after radiotherapy. Thus, multiparametric magnetic resonance imaging (mp-MRI) is performed before radiotherapy and in case of suspected local relapse^7^. mp-MRI could be scheduled early after curative intent treatment, as it may become a potential tool for both PCa treatment effectiveness evaluation and outcome prediction^8^. An early biomarker could allow to adapt long-term follow-up on an individual basis. Several authors have studied the impact of mp-MRI and demonstrated the accuracy of mp-MRI for first diagnosis, local invasion, treatment planning, and relapse diagnosis. However, very scarce data about mp-MRI are available for follow-up in order to predict relapse or PSA value^9^. Initial findings indicate that the kinetic of diffusion-weighted MRI (DWI) may be useful for prediction and early assessment of pathologic response to preoperative radiochemotherapy of rectal cancer, with higher accuracy than volumetric measurements^10^.

MRI guided radiation therapy (MRgRT) becomes available and MRI sequences could also be used as an indicator of tumour response during radiotherapy. There are currently no validated MR biomarkers for prostate radiotherapy. MR images acquired throughout a course of MRgRT could allow the dose distribution to be adjusted based on tumour response^11^. Adaptive dose painting could target the index lesion, where local relapse is most likely to occur; there is currently a paucity of data assessing imaging changes during and directly after treatment^11^. DWI, part of mp-MRI, is considered to be a functional sequencing. Contrary to other anatomical sequencing, such as T1-weighted or T2-weighted, DWI enables quantification assessment. This method of imaging analysis should decrease the inter- and intra-operator variability and correlate with histological characteristics. DWI describes a quantification of the Brownian motion of water molecules and thus may reflect cellular population of tissue^12^. It is also assumed that DWI could be seen as a promising tool for the assessment of EBRT effectiveness in PCa^13^. Different apparent diffusion coefficient (ADC) values between healthy and tumor tissue have been reported, along with an increase in ADC values for tumors after treatment in thirteen patients^13^. Therefore, this ancillary study was designed to evaluate DWI/ADC for tumors at different times before and after treatment as an early predictive biomarker for PCa treated by EBRT.

## Results

### Outcome, PSA value and relapse

All patients completed the entire treatment. With a median follow-up of 49.1 months (range from 38.7–61.6), one patient experienced biochemical relapse 37 months after EBRT. The median rates of PSA at the time of diagnosis, two years and four years after EBRT were 9.29 ng/ml (range from 5.26 to 17.67), 0.68 ng/ml (0.07–2.7), 0.47 ng/ml (0.09–1.39), respectively. All of the patients had decreased PSA since M6, but one patient had a non-significant PSA bounce at M18 (1.01 ng/ml and 1.04 ng/ml respectively at M12 and M18). At the end of the follow-up, eight patients presented a PSA rate below 1 ng/ml including five patients with a PSA below 0.5 ng/ml.

### nADC kinetic

**Figure 1:**
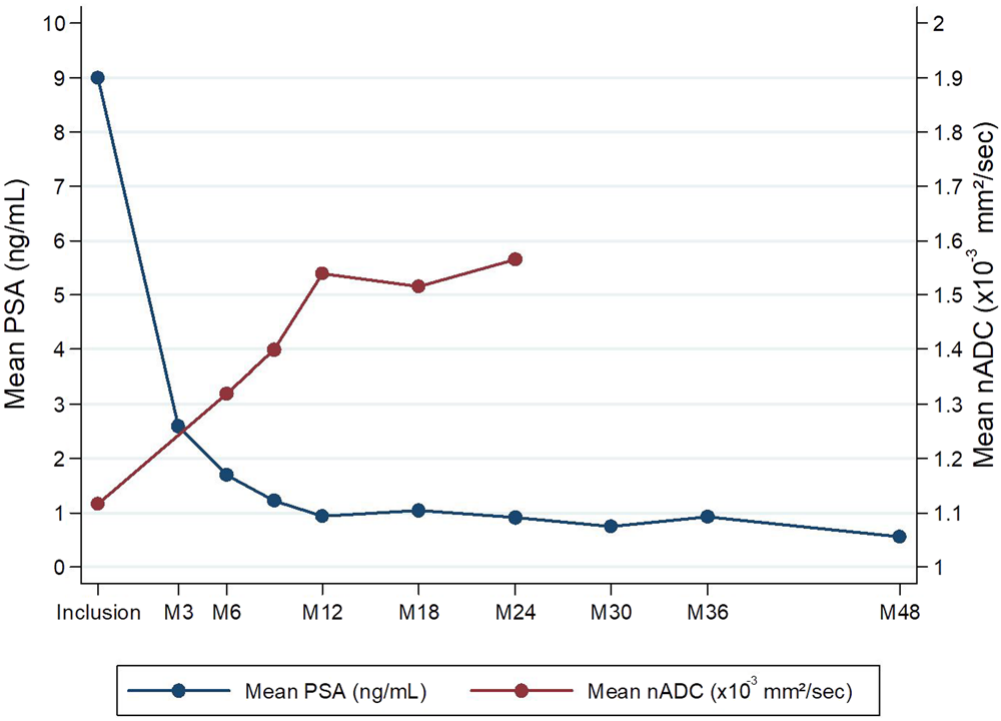
Evolution depending on time of the average PSA and nADC values (Mx = x months after radiotherapy).

Results from median nADC values showed an increase for each mp-MRI from the follow-up. Median nADC was 1.14 × 10^−3^ mm^2^/s (0.49–1.53), 1.16 × 10^−3^ mm^2^/s (0.85–2.59) and 1.52 × 10^−3^ mm^2^/s (1.12–2.18) respectively at the time of diagnosis, 6 months and 12 months after EBRT (Fig. 1). Maximal nADC appeared at M12. nADC values differed significantly between M0 and M12 (p = 0.025, Wilcoxon signed-rank test for paired data). Only one patient presented a decrease of nADC (1.35 × 10^−3^ mm^2^/s and 1.11 × 10^−3^ mm^2^/s at M0 and M12 respectively).

### Statistical analysis

The nADC variation between value at the time of diagnosis (M0) and 12 months after the end of EBRT (M12) was associated with a PSA threshold of 1 ng/ml at M18 (p = 0.034) with a higher increase in nADC at M12 for patients with PSA < 1 ng/mL at M18. The increase in nADC at M6 was correlated with PSA decrease at M18 (p = 0.043), M24 (p = 0.048) and M36 (p = 0.019), and with PSA nadir at M30 (p = 0.048). PSA at M0 seems to be correlated with the increase in percent between nADC value at M0 and M6 after EBRT (p = 0.015). The only patient with a PSA bounce is also the one with decreased nADC between M0 and M12. Only one patient presented with biochemical relapse; this number of events is too small to study the correlation between relapse free survival and nADC.

## Discussion

Treatment effectiveness prediction is a high stake in medicine. D’Amico staging for early PCa is one of the main classifications used to predict the outcome for any treatment plan^6^. Other nomograms are also described to improve the accuracy of the risk of relapse prediction but could present very different results depending on the treatment^14^. In particular, intermediate risk groups present very heterogeneous survival, and thus new pretreatment biomarkers are being studied to better classify this group^15,16^. Few studies have explored post-treatment biomarkers for predicting relapse^17^. Some research also suggests that the PSA threshold at different times of the follow-up after the end of EBRT such as 1 ng/ml or 0.5 ng/ml is correlated with long-term progression-free survival that strengthens the conclusions of our study^18–20^.

The use of multiparametric MRI (mp-MRI) is a paradigm shift in all stages of prostate cancer treatment. DWI is particularly interesting because it may describe tissue characteristics. Some studies have reported a correlation between ADC values, PCa cellular density and sometimes Gleason score^12,21^. The increase of tumor ADC after radiation therapy for PCa was described in a few studies^13,22–24^. Foltz *et al*. found that ADC values are different before and after EBRT with a bigger shift value for tumor tissue^23^. Park *et al*. also described an increase in ADC value after EBRT (p = 0.001) with a maximum reach 1 month after the end of the treatment in eight patients^24^. In details, ADC values before treatment were 1.06, 1.00 and 1.13 × 10^−3^ mm^2^/s in Decker *et al*.^13^, Song *et al*.^22^, and Foltz *et al*.^23^ respectively. After treatment, ADC values were 1.41, 1.61, and 1.30 × 10^−3^ mm^2^/s respectively in the same studies. These results obtained with a standard fractionation are similar to ours whereas a part of the radiotherapy regimen is hypofractionated.

We used nADC to overcome inter-scanner variability. In Zhu *et al*.^25^ the liver nADCs obtained from three different scanners (1.5 and 3 T) were consistent although the liver ADCs varied significantly (p < 0.001). ADC measured at different scanners with different field strengths could not be compared directly but nADC may provide better reproducibility by overcoming these potential issues^25^. The influence of delineation deviations on nADC results are beyond the scope of this study. Moreover, to our knowledge, no study has evaluated it.

In our prospective study the increase in nADC between mp-MRI of M0 and M6 could predict PSA decrease with at least 12 to 30 months in advance. To the best of our knowledge, only one other study has shown a correlation between the variation of tumor ADC and the outcome of patients after prostate cancer radiation therapy. In this retrospective study of a high-risk PCa population treated by EBRT and androgen-deprivation therapy by Liu *et al*. these authors found an impact of tumor ADC value after EBRT showing that early ADC is significantly different between the patients with relapse and the patients free of disease after treatment (AUC = 0.88)^26^. In others cancers, post-treatment ADC or ADC variation seems to be more accurate as a biomarker than only pretreatment value^10,27,28^. Créhange *et al*. described a significant correlation between PSA threshold at 12 months after the end of the treatment by EBRT and choline rate at 6 months in spectroscopy in 24 patients^29^. Some researchers, such as Ginsburg *et al*., have studied radiomics and described prediction models of relapse^30^. These researchers showed that for seven patients over 16, radiomics could predict biochemical relapse with an area under the curve (AUC) of receptor operating curve (ROC) between 0.74 and 0.83 based on pretreatment mp-MRI. These results were since confirmed by Gnep *et al*. on a cohort of 74 patients with high-risk PCa but here again in a retrospective manner contrary to our study^31^.

Our study presents limits, including small number of patients and experimental fractionation. However, the strength of our study is comparable to that of similar studies and its follow-up is longer^13,23,24^. Contrary to previous studies, none of our patients were treated by androgen deprivation, which is known to lower tumor ADC value. In conclusion nADC values between pretreatment and follow-up MRI was modified soon after radiation therapy for prostate cancer in our series. Early increase in nADC was correlated with long term PSA kinetic and nadir. To date our series is the only prospective series that has shown this correlation. If validated, these results could open the door to tailored treatment or follow-up. Indeed in a next future images acquired throughout a course of MRgRT could allow the dose distribution to be adjusted based on tumour response^11^. Thanks to ADC early kinetic during radiotherapy, it could permit to adapt dose for patient with worse outcome prediction but also for intra-prostatic dominant lesion. Adaptive dose painting could target the index lesion or a tumour with little variation in ADC during treatment and potentially less sensitive to radiation therapy. These results could lead to design studies using new MR-Linac facilities^11^. Further studies are needed before using DWI as an early biomarker of treatment effectiveness.

## Materials and Methods

### Patients’ and tumors’ characteristics

Twelve consecutive patients, treated in our institution between June 2010 and June 2013 within the frame of a multicenter phase II trial (CKNOpro)^32,33^ were included. The design of this study was approved by the ethical commission “Comite de Protection des Personnes Nord Ouest IV”. All methods were carried out in accordance with relevant guidelines and regulations. All experimental protocols were approved by the national committee “Agence française de securite sanitaire des produits de sante”. Written informed consent was obtained from all patients included in the study. Informed written consent was obtained from all subjects. All patients presented with histologically proven PCa with intermediate risk according to d’Amico classification. Population characteristics are described in Table 1.

**Table 1:** Population Characteristics.

N°	Gleason (X + X)	Tumor localization	TNM	nADC M0	PSA M0	MRI (T)
(1)	7 (4 + 3)	PZ	T2a	1.09	5.6	1.5
(2)	7 (4 + 3)	PZ	T2b	0.49	5.26	3
(3)	6 (3 + 3)	PZ	T1c	1.53	9.72	3
(4)	6 (3 + 3)	PZ	T2a	0.76	9.88	3
(5)	7 (3 + 4)	PZ	T1c	1.31	11.55	3
(6)	7 (4 + 3)	CZ	T2a	1.46	6	3
(7)	6 (3 + 3)	CZ	T2a	1.36	12.02	3
(8)	7 (4 + 3)	PZ	T2a	1.10	5.33	3
(9)	6 (3 + 3)	NA	T2b	1.19	5.96	3
(10)	7 (3 + 4)	TZ	T1c	1.35	10.07	3
(11)	6 (3 + 3)	PZ	T1c	1.04	17.67	3
(12)	7 (3 + 4)	NA	T2b	0.70	8.86	3

N°: Identification number of patients; Gleason: Gleason score (Addition of the two most frequent gleason score population); TNM: TNM staging; MRI: type of MRI machine; PSA: prostate specific antigen in ng/ml; M0: Time of diagnosis; nADC: normalized apparent coefficient value in 10^−3^ mm^2^/s; –: no value available; Tumor localization: PZ for peripheral zone, TZ for transition zone and CZ for central zone. NA if tumor invaded more than one localization; Magnetic Resonance Imaging field (Tesla).

### Treatments modalities

This study evaluated the effectiveness and toxicity of an EBRT combination of 46 Gray (Gy) with normal fractionation followed by a hypofractionated stereotactic boost (3 fractions of 6 Gy). The first part of the treatment was delivered using 3D conformal radiotherapy (3DCRT) or intensity modulated radiation therapy (IMRT) associated with image-guided radiotherapy (cone beam or megavoltage computed tomography). Next, stereotactic body radiotherapy (SBRT) was delivered using Cyberknife (Accuray, Inc., Sunnyvale, CA). Treatment volume included prostate and the first part of seminal vesicles and prostate only for EBRT and SBRT respectively. Prostate volume was delineated using planning MRI registration based on intraprostatic fiducials. None of these patients had received hormonal therapy before or during radiotherapy.

### Follow-up

All the patients underwent an mp-MRI exam, biological evaluation (PSA) and clinical examination before treatment (M0). After treatment, the same evaluation was scheduled at three months then every six months until 36 months after treatment (M6-M36) and annually during two additional years (M48 and M60). Biochemical relapse was considered if PSA rate increased over nadir + 2 ng/ml following Phoenix criteria. To date data with a median follow-up of 49 months are available.

### MRI protocol

Mp-MRI used a 1.5 or 3 T MRI (both Discovery, GE medical system) and included at least a T1 weighted, T2w, a Dynamic contrast enhanced (DCE) and DWI sequences for each time. DWI sequencing was natively acquired with two or three b-values: 0, 1000, 2000 mm^2^/s (2000 mm^2^/s for 3 T MRI only). ADC maps were calculated using an homemade software previously described using all of the b-values for each exam^12^. Usually, two b-values are used to estimate ADC because the relationship between b-values and ADC values is mono-exponential. In this study, because certain patients had multi b-values, ADC value was computed as the Powell optimization algorithm of ADC = argmin(Σ_(i = 1)^N^ [S_i − S_0 e^(−ADC*b_i)^]2) n. “n” is the number of b-values used for the equation. Mean ADC in mm^2^/s (nADC) were normalized to ADC results from obturator muscle. A region of interest (ROI) was delineated within the obturator muscle, in an area which was not irradiated. The tumor ADC value was divided by the muscle ADC to obtain nADC. Sequencing characteristics and acquisition parameters are described in Table 2.

**Table 2:** MRI and sequencing characteristics.

Parameters	Echo-planar DWI 1.5 T	Echo-planar DWI 3 T
TR (ms)	3200	4000
TE (ms)	95	76
Flip angle (degrees)	90	90
Matrix (frequency 3 phase)	192 × 192	256 × 256
N° of acquired signals	3	3
Field of view (mm)	340 × 340	240 × 240
Acquired pixel size (mm)	1.3 × 1.3	1.5 × 1.5
Slice thickness (mm)	3	3.5

DWI: Diffusion-Weighted Imaging; TR: Repetition Time; TE: Echo Time.

### Delineation methods and statistical analysis

Gross tumor volume ROI was delineated by a radiation oncologist helped by experimented radiologist. ROI delineation used data from all the sequencing of the mp-MRIs and biopsy reports. Only tumors seen on mp-MRIs according to ESUR guidelines were delineated^34^. While drawing ROIs, the largest possible part of the tumor was included. Pre-therapeutic delineation was reported on a follow-up MRI using rigid registration. A visual control was used to ensure concordance of the prostate and tumor contours. If necessary, ROIs were placed in the same location where pre-therapeutic ROIs were drawn with regards to anatomic landmarks and respective image findings for consistency.

Because only one patient presented with relapse, imaging quantification was correlated with PSA at different times after the treatment. Association between mp-MRI and PSA or PSA kinetic between diagnosis and follow-up was evaluated using the test of nullity of the Spearman correlation coefficient. Association between mp-MRI and PSA categorized as binary value using the cutoff 1 ng/mL or 0.5 ng/mL was performed using Wilcoxon Mann-Whitney non-parametrical test. All of the analyses were performed using STATA software (StataCorp LLC, Texas, Us). A p-value < 0.05 was considered to be statistically significant.
